# Real-Time Brain Monitoring by Near-Infrared Spectroscopy Predicts Neurological Outcome after Cardiac Arrest and Resuscitation in Rats: A Proof of Concept Study of a Novel Prognostic Measure after Cardiac Arrest

**DOI:** 10.3390/jcm11010131

**Published:** 2021-12-27

**Authors:** Ryosuke Takegawa, Kei Hayashida, Tai Yin, Rishabh C. Choudhary, Santiago J. Miyara, Houman Khalili, Muhammad Shoaib, Yusuke Endo, Emesto P. Molmenti, Lance B. Becker

**Affiliations:** 1 Feinstein Institutes for Medical Research, Northwell Health, Manhasset, NY 11030, USA; RTakegawa@northwell.edu (R.T.); tyin@northwell.edu (T.Y.); rchoudhary1@northwell.edu (R.C.C.); smiyara@northwell.edu (S.J.M.); hkhalili@northwell.edu (H.K.); mshoaib1@northwell.edu (M.S.); yendo@northwell.edu (Y.E.); lance.becker@northwell.edu (L.B.B.); 2 Department of Emergency Medicine, North Shore University Hospital, Northwell Health System, Manhasset, NY 11030, USA; 3 Department of Traumatology and Acute Critical Medicine, Graduate School of Medicine, Osaka University, Osaka 565-0871, Japan; 4 Zucker School of Medicine at Hofstra/Northwell, Hempstead, NY 11549, USA; emolmenti@northwell.edu; 5 Department of Surgery, North Shore University Hospital, Northwell Health, Manhasset, NY 11030, USA

**Keywords:** near-infrared spectroscopy, cerebral oxygen saturation, cardiac arrest, electroencephalography, brain oxygen consumption

## Abstract

Clinical studies have demonstrated that dynamic changes in regional cerebral oxygen saturation (rSO_2_) after cardiac arrest (CA) and cardiopulmonary resuscitation (CPR) have a role in predicting neurological outcomes after the return of spontaneous circulation (ROSC). Our study evaluated whether the timing of rSO_2_ decline shortly after CPR reflects the severity of brain injury in a rat model of CA. Rats were subjected to different durations of asphyxia to produce variable severities of brain injury, due to CA. Time from ROSC to achieving the initial minimum rSO_2_ was defined as T_nadir_. A T_nadir_ cut-off of 24 min had optimal sensitivity and specificity for predicting good neurological outcomes at 72 h after ROSC (AUC, 0.88; sensitivity, 89%; specificity, 86%; *p* < 0.01). Immunohistochemistry at 72 h post-CA revealed that the number of Fluoro-Jade B positive degenerating neurons in the hippocampus CA1 sector were markedly higher in animals with T_nadir_ > 24 min than that in animals with T_nadir_ ≤ 24 min. There was no difference in the gene expressions of cytokines and mitochondrial fission proteins in the brain at 2 h after ROSC between rats with T_nadir_ > 24 min and with T_nadir_ ≤ 24 min. In conclusion, T_nadir_ can be a novel predictor of good neurological outcomes after CA/CPR.

## 1. Introduction

Sudden cardiac arrest (CA) is a major public health problem globally, which accounts for over 355,000 emergency medical, service-assessed, out-of-hospital cardiac arrest (OHCA) cases in the United States [1]. Although several studies have reported the trend of improved survival rates and favorable neurological outcomes in patients with OHCA who received cardiopulmonary resuscitation (CPR) [1,2,3], the global rate of hospital discharge, with intact neurocognitive function among these patients is still low [2,4]. Adopting more effective methods of real-time brain monitoring may improve cognitive outcomes after return of spontaneous circulation (ROSC) [5] and optimize therapeutic interventions; however, no timely and reliable single prognostication marker is available for patients who remain comatose after CA/CPR [6].

Recently, regional cerebral oxygen saturation (rSO_2_), measured using near-infrared spectroscopy (NIRS), has emerged as a monitoring system for predicting the probability of ROSC and/or favorable neurological outcomes after successful CPR [7,8,9]. More recent studies have suggested that dynamic assessments of rSO_2_, obtained throughout resuscitation, are more appropriate than single-time assessments for predicting the outcomes in patients with OHCA [9,10,11,12,13]. Although the role of temporal changes in rSO_2_ shortly after ROSC in the neurological prognosis remains undetermined, some clinical studies have reported that rSO_2_ shortly after ROSC shows a biphasic pattern, such that lower rSO_2_ shortly post-ROSC is associated with favorable neurological outcomes [12,14]. These observations suggest that the duration in which rSO_2_ reaches the initial lowest value (“nadir”) may reflect the duration in which brain activity and oxygen metabolism would resume after ROSC. However, the prognostic implication of temporal changes in rSO_2_ after ROSC, that can be measured in a timely and non-invasive manner, is unclear and remains to be investigated.

Therefore, in the present study, we elucidated the role of temporal changes in rSO_2_, shortly after ROSC, in: (1) assessing the severity of brain damage and (2) CA-related pathophysiological changes, as well as (3) predicting neurological outcomes after CA/CPR in rats.

## 2. Materials and Methods

### 2.1. Experimental Animals

Adult male Sprague-Dawley rats (435 ± 26.7 g, 12–16 weeks old; Charles River Laboratories, Wilmington, MA, USA) were used in this study. All experiments were performed in accordance with the National Institutes of Health guidelines for the use of experimental animals. The animals were housed in a rodent facility, under a 12:12-h light:dark cycle, and had free access to food and water. 

After being anesthetized with 4% isoflurane, the animals were intubated with a 14-gauge plastic catheter (Surflo, Terumo Medical Corporation, Somerset, NJ, USA), mechanically ventilated, and then surgically prepared under anesthesia (2% isoflurane), as described previously [15]. End-tidal carbon dioxide was maintained at 40 ± 5 mmHg during the experiment, due to the adjustment of respiratory rate (RR) and tidal volume (TV). These settings were adjusted, within the range of 35–50 per min of RR and 3.0–5.0 mL of TV. Microcatheters (PE-50, Becton Dickinson, Franklin Lakes, NJ, USA) were inserted into the left femoral artery and the left femoral vein for monitoring blood pressure and drug administration, respectively. After cannulation, heparin (300 U) was injected via the femoral vein. A customized brain oximeter was attached to the rats’ bare heads, to monitoring rSO_2_ for 2 h during the experimental period. Esophageal temperature was maintained at 36.5 ± 0.5 °C, using a thermostatically regulated heating pad and heating lamp during the experiment. Blood pressure and needle-probe electrocardiogram monitoring data were recorded and analyzed using a personal computer-based data acquisition system.

### 2.2. Monitoring by Near-Infrared Spectroscopy

A brain oximeter, TOS-QQ^®^ (TOSTEC, CO., Tokyo, Japan), was used in this study. This device measures oxygen saturation in the brain every second, based on the Beer–Lambert law, using two different wavelengths of near-infrared LED light, i.e., 770 nm and 870 nm, which are the wavelengths specific for the absorbance of deoxyhemoglobin and oxyhemoglobin, respectively [13]. As the source–detector distance is 10 mm, near-infrared LED light is estimated to pass through the skin to a depth of approximately 7–8 mm, suggesting that it can measure the maximum depth of the hippocampus in rats. 

### 2.3. Definition of T_nadir_ as a Predictive Marker for Neurological Outcome after CA/CPR

Using the rSO_2_ data extracted every 1 min, we measured the duration from ROSC to the time point at which rSO_2_ reached the first and lowest value after ROSC, which was predefined as T_nadir_, based on our pilot study. Our pilot study, using a rat model of asphyxia-induced CA for 6–12 min, found that rSO_2_ always increased quickly and achieved the highest value within 15 min after ROSC, following which, it decreased gradually within approximately 15–60 min, according to the individual’s condition. Moreover, from the clinical viewpoint, we consider that T_nadir_ should be determined in real time. Taken together, the first and lowest rSO_2_ value after ROSC were determined, if the following conditions were met: (1) rSO_2_ decreased by more than 3% from the highest rSO_2_ shortly after ROSC, and (2) rSO_2_ increased by more than 1%, or remained unchanged for a continuous 3 min. According to the criteria, we defined the duration from ROSC to the time point at which rSO_2_ achieved the first and lowest value as T_nadir_.

### 2.4. Study Design

**Figure 1:**
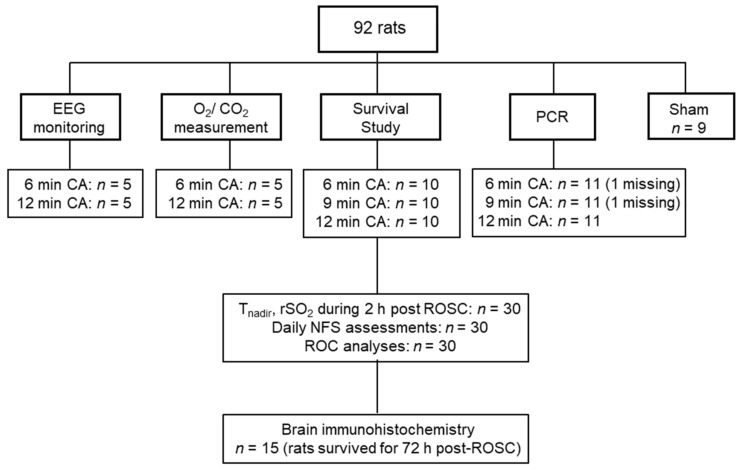
Flow diagram of the experimental groups. Data for two samples (in 6 and 9 min CA groups) in the PCR analysis were missing, due to technical failures. Brain samples from sham-operated rats were used for brain immunohistochemistry (*n* = 2) and PCR analysis (*n* = 7). EEG; electroencephalography, O_2_; Oxygen, CO_2_; carbon dioxide, PCR; polymerase chain reaction, CA; cardiac arrest, rSO_2_; regional cerebral oxygen saturation, ROSC: return of spontaneous circulation, NFS; neurological function score, ROC analyses; receiver operating characteristic analyses.

Rats were subjected to different durations of asphyxia to produce variable severities of brain injury due to CA. Four separate protocols were used in this study (Figure 1).

In Experiment 1, to test the hypothesis that a longer CA duration results in a longer cerebral electrical recovery latency post-ROSC, electroencephalography (EEG) was monitored in rats subjected to 6 or 12 min CA (*n* = 5 per group). 

In Experiment 2, to test if CA duration is related to cerebral oxygen metabolism post-ROSC, we measured the oxygen/carbon dioxide levels by using the arterial and venous blood gas analyses in rats subjected to 6 or 12 min CA (*n* = 5 per group).

In Experiment 3, we aimed to assess the association of T_nadir_ with neurofunctional status at 72 h after CA/CPR. Rats were subjected to 6, 9, or 12 min of CA, to induce various types of brain damages after CA/CPR, as previously reported [16]. To minimize variability, we assigned animals to 6, 9, or 12 min of CA, on the basis of similar weight, age, and, when possible, delivery date (*n* = 10 per group). Using rats survived for 72 h, the brains were obtained and used for immunohistochemistry.

In Experiment 4, to determine the mechanisms associated with the predictive role of T_nadir_, we assessed the association of T_nadir_ with mRNA levels of genes encoding proinflammatory cytokines and mitochondrial fission-related proteins in the brains of animals at 2 h post-ROSC.

### 2.5. Rat Model of Asphyxial CA and CPR

Cardiac arrest and CPR in rats were performed, as previously described, with minor modifications [15,17,18]. Briefly, prior to the induction of asphyxial CA, animals were mechanically ventilated, with a fraction of inspired O_2_ (F_I_O_2_) of 0.25, and anesthesia was maintained with 2% isoflurane during surgical procedures. Asphyxia was induced by intravenous vecuronium bromide (2 mg/kg), followed by switching off the ventilator, and discontinuation of isoflurane. Cardiac arrest was defined by a mean arterial pressure below 20 mmHg. After asphyxia and CA, mechanical ventilation was restarted at an F_I_O_2_ of 1.0, and manual chest compressions were performed at a rate of 240–300 per min. The investigator who provided finger–chest compressions was not blinded to the experimental groups. At 30 s, after the beginning of chest compressions, a 20 μg/kg bolus of epinephrine was administered, and chest compressions were continued. ROSC was defined as the return of supraventricular rhythm, with a mean arterial pressure > 60 mmHg. Animals were mechanically ventilated with an F_I_O_2_ of 1.0 for the first 105 min after ROSC. Thereafter, F_I_O_2_ was gradually reduced to 0.6, 0.3, and 0.25 at 110, 115, and 120 min, respectively, followed by disconnection from mechanical ventilator and extubation. Arterial blood pressure, rSO_2_, electrocardiogram recordings, and esophageal temperature were monitored for 2 h. No inotropic agent was administered. After a recovery period of 2 h, the animals were weaned from the ventilator, all vascular catheters and tracheal tubes were removed, and surgical wounds were sutured. For the survival study (Experiment 3), animals were returned to their cages, with easily accessible food and water, and observed in a rodent facility, with a controlled room temperature of 22 °C. Buprenorphine (0.02 mg/kg) was injected subcutaneously daily to relieve the animals from pain, due to surgery, during the recovery period. The survival time after CA/CPR was recorded up to 72 h. Sham-operated rats were administered with anesthesia, underwent surgery, and received ventilation, but no CA.

### 2.6. Electroencephalography Monitoring

In Experiment 1, EEG after ROSC was monitored, as reported previously [19]. Briefly, before intubation and cannulation procedures, animals underwent implantation of EEG electrodes bilaterally under isoflurane, using a stereotaxic apparatus (Stoelting, Chicago, IL, USA). Each animal had five screw electrodes (Plastics One, Roanoke, VA, USA) cortically implanted 2 mm lateral and 2 mm anterior or posterior to the bregma. In addition, a ground electrode was placed at 3 mm lateral on the right and 9 mm anterior to the bregma in the midline. EEGs were recorded using an Intan RHS Stim/Recording 16 channel recording controller (Intan Technologies, Los Angeles, CA, USA) during the baseline, asphyxial CA, resuscitation, and after ROSC. Raw EEG signals were used to determine the electrical activity. In addition, the durations between ROSC and onset of both EEG amplitude activity (defined as >5% of the basal value with no EEG activity), as well as continuous background EEG as markers of brain’s electrical recovery after CA/CPR, were also recorded [20]. Continuous background EEG activity was defended as continuous EEG activity/burst without low amplitude activity (suppression, <10 µV). EEG analysis was performed offline using MATLAB 7.0 (MathWorks, Inc., Natick, MA, USA) after the experiment was completed.

### 2.7. Assessment of Oxygen Consumption and Carbon Dioxide Production in the Brain

In Experiment 2, the right jugular vein and right carotid artery were cannulated (PE-50) for blood collection, and blood gases were analyzed at baseline, 20 min, and 60 min after ROSC, in rats subjected 6 or 12 min CA (*n* = 5 per group). Cerebral O_2_ consumption was calculated as the difference between O_2_ content of the carotid artery (CaO_2_) and jugular vein (CjvO_2_): CaO_2_ − CjvO_2_ = (1.36 × Hemoglobin (g/dL) × (arterial venous oxygen saturation (SaO_2_] − jugular bulb venous oxygen saturation (SjvO_2_])/100) + (arterial oxygen tension (PaO_2_) − jugular bulb venous oxygen tension (PjvO_2_]) × 0.0031 [21,22]. Cerebral CO_2_ production was calculated as the difference between CO_2_ content of the jugular vein (CjvCO_2_) and carotid artery (CaCO_2_). The difference in CO_2_ content was calculated using the McHardy equation: CjvCO_2_ − CaCO_2_ = 11.02 ((PjvCO_2_) 0.396 − (PaCO_2_) 0.396) − (15 − Hb) 0.015 × (PjvCO_2_ − PaCO_2_) − (95−SaO_2_) × 0.064 [23].

### 2.8. Assessment of Neurological Function

For the survival study (Experiment 3), neurological function score (NFS) was evaluated daily after CA/CPR by an investigator blinded to the experimental groups, using a previously reported neurofunctional scoring system with minor modification [24]. Specifically, original scoring system provided the percentage of the deficit score, but we showed the percentage of total score. Eventually, dead or brain-dead rats were scored at 0 points. The resuscitated animals were classified into binary groups using the NFS cut-off for good (NFS ≥ 60) and poor (NFS < 60) outcomes at 72 h post-ROSC, as previously described [16].

### 2.9. Measurement of Interleukin (IL)-6 Levels in the Plasma

In Experiment 3, plasma samples were obtained at baseline and 60 min after ROSC. The levels of plasma IL-6 were measured using ELISA kits (Quantikine^®^ ELISA, R&D Systems, Inc., Minneapolis, MN, USA), according to the manufacturer’s instructions.

### 2.10. Brain Immunohistochemistry

In Experiment 3, brain samples were harvested from rats survived for 72 h. Neuronal degeneration was evaluated using Fluoro-Jade B (FJB) staining, as described previously [25,26]. Briefly, following the 72-h survival in rats subjected to 6–12 min CA, animals were anesthetized with isoflurane, perfused with saline via the left ventricle, and then decapitated. The brains were fixed with 4% paraformaldehyde at 4 °C. Tissues were embedded in OCT Compound (Tissue-Tec, Sakura Finetek USA, Inc., Torrance, CA, USA), frozen in liquid nitrogen, cut serially with 10 µm thickness in a cryostat, and collected on glass slides. Three microscopic fields (at 40× magnification) were selected from the cerebral cortex, hippocampus, and caudoputamen by an investigator who was blinded to the experimental groups. The investigator selected arbitrary points that were considered to show the representative images of the entire area of each brain region. The number of FJB-positive cells was counted by an investigator who was blinded to the experimental groups (*n* = 6, 4, and 5 for 6, 9, and 12 min CA, respectively). Images were captured using a BZ-X800 fluorescence microscope (Keyence Corporation of America, Elmwood Park, NJ, USA). 

### 2.11. Measurements of Gene Expressions

In Experiment 4, Total RNA was extracted from the whole brain of rats 2 h after CA/CPR in rats subjected to 6–12 min CA, using RNeasy Plus Mini Kit (Qiagen, Hilden, Germany), and cDNA was synthesized using High-Capacity cDNA Reverse Transcription Kit (Thermo Fisher Scientific, Waltham, MA, USA) (*n* = 11 per group). The mRNA levels of interleukin (IL)-1β, IL-6, tumor necrosis factor (TNF)-α, high mobility group box 1 (HMGB1) protein, dynamin-1-like (Dnm1L) protein, mitochondrial fission 1 (FIS1) protein, mitochondrial elongation factor 1 (MIEF1) protein, and GAPDH were measured in real-time PCR, using the TaqMan assay (Thermo Fisher Scientific). The mRNA expression levels were normalized against the levels of GAPDH mRNA in the same sample. Relative mRNA levels in each sample were evaluated using the ΔΔCT method. Data are presented as fold changes relative to the control group. The assay IDs and primer sequences for each gene are listed in Table S1.

### 2.12. Statistical Analysis

Continuous variables are presented as means ± standard deviation (SD). An unpaired two tailed Student’s *t*-test or Mann-Whitney U test was used to compare two independent groups for continuous variables, according to normality. For multiple comparisons, two-way repeated measures analysis of variance (ANOVA), followed by Sidak correction for post-hoc comparisons, was used. Kaplan–Meier analysis and the log-rank test were used to calculate survival rates. Spearman’s correlation coefficients (r) were calculated to evaluate the correlation between T_nadir_ and neurological outcomes 72 h after ROSC. In Experiment 3, the predictive accuracy of T_nadir_ was determined by receiver operating characteristic (ROC) curve analysis (specifically, area under the curve [AUC]). ROC curve analyses determined the optimal cutoff value for differentiating between good and poor neurologic outcomes at 72 h after CA/CPR. The AUCs between T_nadir_ and IL-6 were compared using a paired comparison, originally described by Hanley and McNeil [27]. Differences with two-sided *p*-value < 0.05 were considered statistically significant. All statistical analyses were performed using GraphPad Prism, version 8.3.0 (GraphPad Software, Inc., San Diego, CA, USA), and JMP Statistical Software, version 14.3.0 (SAS Institute, Cary, NC, USA).

## 3. Results

This section may be divided by subheadings. It should provide a concise and precise description of the experimental results and their interpretation, as well as the experimental conclusions that can be drawn.

### 3.1. The Impact of Different CA Durations on Brain Recovery Time and Cerebral Metabolism after ROSC

**Figure 2:**
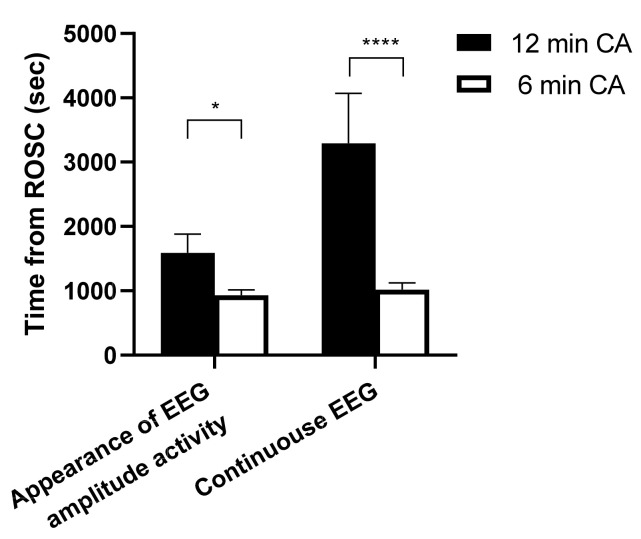
Brain electrical recovery after cardiac arrest/cardiopulmonary resuscitation. The duration of both the appearance of the first EEG amplitude activity and continuous EEG activity were significantly longer in the 12 min CA group than in the 6 min CA group. Data are presented as mean ± SD. *n* = 5 each group. * *p* < 0.05, **** *p* < 0.0001. CA: cardiac arrest.

**Figure 3:**
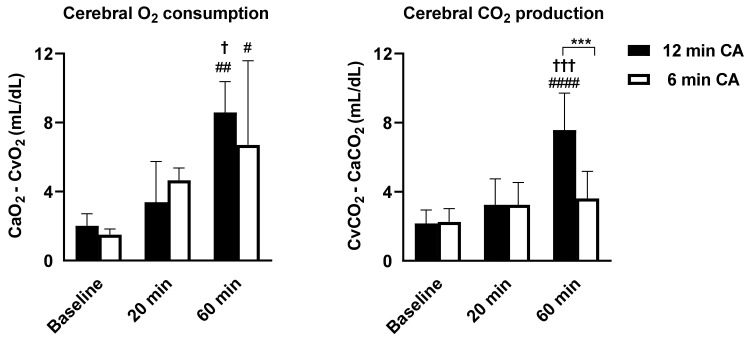
Time-dependent changes in cerebral oxygen metabolisms between 6 and 12 min cardiac arrest and cardiopulmonary resuscitation. (**Left**) Cerebral O_2_ consumption and (**right**) cerebral CO_2_ production at baseline, 20 min, and 60 min after the return of spontaneous circulation in each group; *n* = 5 each group. Data are presented as mean ± SD. ^#^ *p* < 0.05, ^##^ *p* < 0.01, ^####^ *p* < 0.0001 vs. at baseline in the same group, ^†^ *p* < 0.05, ^†††^ *p* < 0.001 vs. at 20 min in the same group, *** *p* < 0.001 between each group.

First, to confirm whether a longer duration of CA affects brain recovery time and cerebral metabolism, we measured electrical activity and cerebral oxygen consumption after CA/CPR in the brain in CA models of two different severities (6 min CA vs. 12 min CA). Animals subjected to 12 min CA had markedly longer duration from ROSC to the onset of both appearance of EEG amplitude activity (12 min CA vs. 6 min CA, 1590 ± 292 s vs. 933 ± 82 s, *p* = 0.048) and continuous EEG (3295 ± 774 s vs. 1019 ± 105 s, *p* < 0.0001) (Figure 2). In both 6 min and 12 min CA groups, cerebral O_2_ consumption at 60 min post-ROSC was significantly higher than that at baseline. While animals subjected to 6 min CA did not exhibit a substantial difference in cerebral CO_2_ production over time, animals subjected to 12 min CA showed a marked increase in cerebral CO_2_ production, compared to that at baseline and 20 min post-ROSC (Figure 3). In addition, animals subjected to 12 min CA had markedly larger cerebral CO_2_ production than animals subjected to 6 min CA (7.57 ± 2.14 mL/dL vs. 3.61 ± 1.57 mL/dL, *p* = 0.0006). These observations suggest that prolonged CA was associated with both delayed electrical recovery of the brain and greater changes in cerebral oxygen metabolism.

### 3.2. T_nadir_ by Real-Time NIRS Monitoring Predicted a Short-Term Neurofunctional Status after CA/CPR

**Figure 4:**
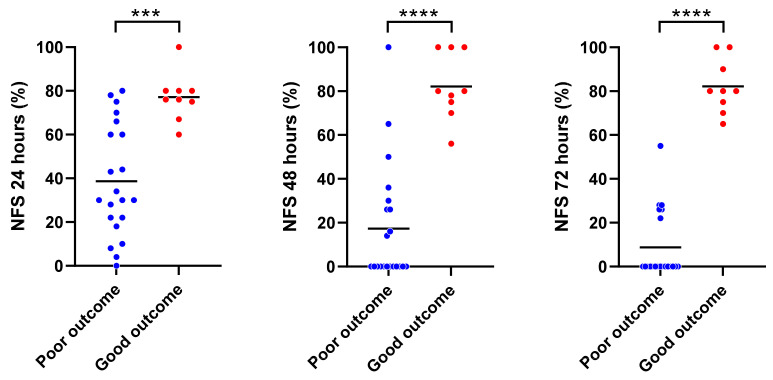
Daily neurological functional scores (NFS) in the good and poor outcome groups after the return of spontaneous circulation (ROSC). The good neurological outcome group exhibited markedly higher NFS at 24, 48, and 72 h post-ROSC. Lines indicate mean; *** *p* < 0.001, **** *p* < 0.0001.

**Figure 5:**
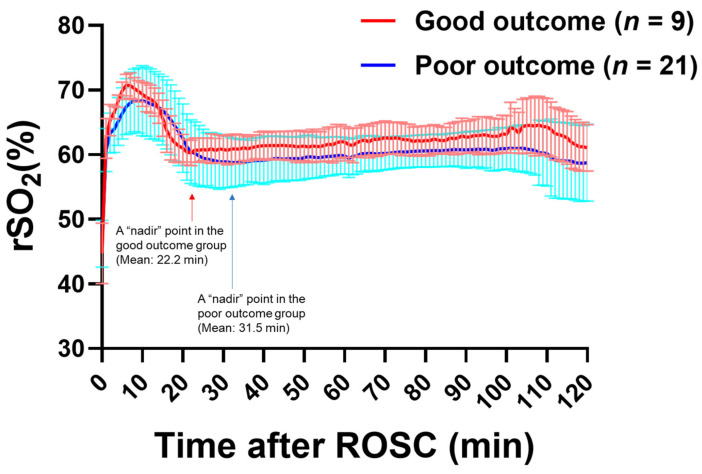
Serial change in regional cerebral oxygen saturation (rSO_2_) during first 2 h after the return of spontaneous circulation (ROSC) in the good (a red line) and poor outcome (a blue line) groups. The 72-h survived rats were classified into binary groups using the NFS cut-off for good (NFS ≥ 60) and poor (NFS < 60) outcomes at 72 h post-ROSC. The rSO_2_ value increased rapidly with ROSC and decreased to a “nadir” level after reaching the peak level. After reaching the nadir, the rSO_2_ value increased gradually, showing a biphasic pattern. Fraction of inspired oxygen (F_I_O_2_) was maintained at 1.0 after initiation of CPR. F_I_O_2_ was gradually reduced to 0.6, 0.3, and 0.25 at 110, 115, and 120 min, respectively, followed by disconnection from mechanical ventilator and extubation. There were no significant differences in rSO_2_ over time between the good and poor neurological outcome groups; *p* = 0.92 for the two-way analysis of variance. The red and blue arrows indicate the “nadir” of rSO_2_ in the good and poor outcome groups, respectively. Data are presented as mean ± SD.

**Figure 6:**
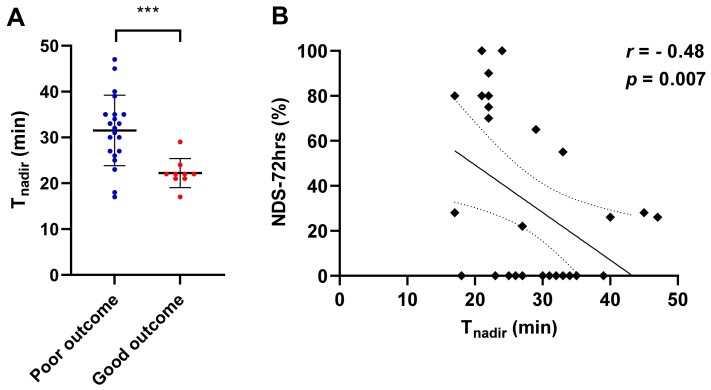
The association of T_nadir_ with neurological function score (NFS) at 72 h post-return of spontaneous circulation. (**A**) Animals in the good outcome group exhibited a shorter T_nadir_ than those in the poor outcome group (22.2 ± 3.2 min vs. 31.5 ± 7.7 min, *** *p* < 0.001). Closed circles indicate T_nadir_ in each individual. The tentacles indicate mean ± SD in each group. (**B**). There was a significant negative correlation between T_nadir_ and NFS at 72 h after return of spontaneous circulation (r = −0.48; *p* = 0.007). Solid line indicates a correlation coefficient (r). Dashed lines show 95% confidence intervals.

In total, 30 rats were randomly subjected to 6, 9, or 12 min of CA, followed by CPR. There were no significant differences in the baseline characteristics of animals among the three different CA duration groups (Table S2). Survival rates at 72 h after ROSC were 60%, 40%, and 50%, and NFS at 72 h after ROSC were 53 ± 46, 27 ± 35, and 13 ± 14 in 6 min, 9 min, and 12 min CA groups, respectively. To assess the role of T_nadir_ in predicting neurofunctional status after CA/CPR, we compared T_nadir_ between post-arrest animals with good neurological outcomes and those with poor neurological outcomes. The baseline characteristics and perioperative parameters of the animals in good and poor neurological outcome groups are shown in Table 1. Significant differences were observed in heart rate and body temperature at baseline, time to cardiac arrest, and heart rate at 20 min after ROSC between the two groups. The survival rate was markedly higher in the good neurological outcome group than in the poor neurological outcome group (100% vs. 28.6%, log-rank *p* = 0.001). NFS at 24, 48, and 72 h after ROSC in good vs. poor neurological outcome groups are shown in Figure 4. The serial changes in rSO_2_ within the first 2 h after ROSC in each group are shown in Figure 5. No differences were observed in rSO_2_ values at any time point over the first 2 h after ROSC between the groups (*p* = 0.92). The mean T_nadir_ of all post-arrest animals was 28.7 ± 1.4 min. Survived animals in the good outcome group had markedly shorter T_nadir_ than those in the poor outcome group (22.2 ± 3.2 min vs. 31.5 ± 7.7 min, *p* < 0.001, Figure 6A). Furthermore, a significant negative correlation between Tnadir and the NFS 72 h after ROSC (r = −0.48, *p* = 0.007, Figure 6B) was observed. However, there was no significant correlation between survival time and T_nadir_ (r = −0.30, *p* = 0.11).

### 3.3. Predictive Value of T_nadir_ on Good Neurological Outcome Was Superior to That of Plasma IL-6 Level at 1 h after ROSC

**Figure 7:**
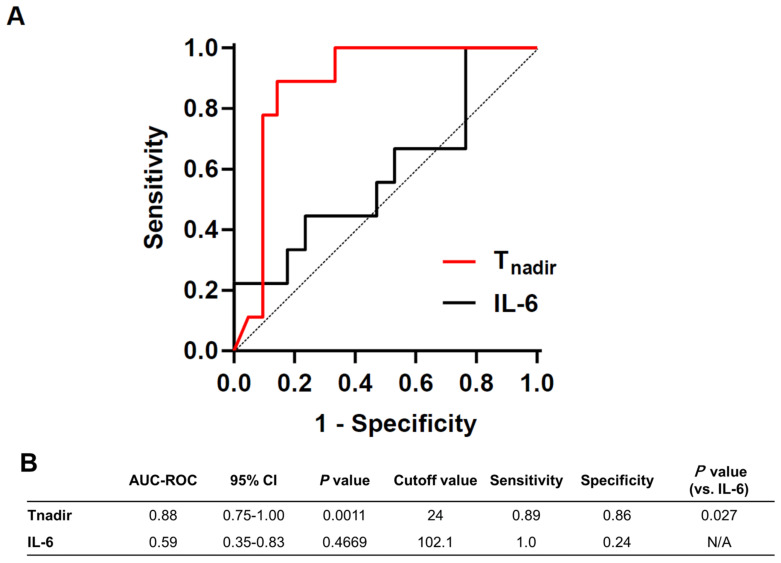
ROC analyses. (**A**) The ROC-AUC revealed that a T_nadir_ cut-off of 24 min provided optimal sensitivity and specificity for predicting good neurological outcomes (AUC, 0.88; 95% CI, 0.75–1.0; sensitivity, 88.9%; specificity, 85.7%; *p* = 0.001). (**B**) The AUC of plasma IL-6 levels at 1 h after return of spontaneous circulation, for predicting good neurological outcomes, was 0.59 (95% CI, 0.35–0.83; *p* = 0.47). The AUC of T_nadir_ was significantly superior to that of plasma IL-6 (*p* = 0.027). AUC: area under the curve, CI: confidence interval, IL-6: interleukin 6.

Since plasma IL-6 levels after ROSC have been shown to be independently associated with poor neurological outcomes in patients with CA [28,29,30,31], we measured plasma IL-6 levels shortly after CA. Cardiac arrest led to increased IL-6 levels at 1 h post-ROSC (baseline vs. 1 h post-ROSC, 92.1 ± 13.7 pg/mL vs. 120.5 ± 42.5 pg/mL, *p* < 0.0001). There was no significant correlation between survival time and IL-6 levels (r = 0.17, *p* = 0.40). ROC curve analyses were performed to assess the accuracy of T_nadir_ and plasma IL-6 levels in differentiating between good and poor neurological outcomes at 72 h post-ROSC. ROC analyses revealed that a T_nadir_ cut-off of 24 min provided optimal sensitivity and specificity for predicting good neurological outcomes (AUC, 0.88; 95% confidence interval (CI), 0.75–1.0; sensitivity, 88.9%; specificity, 85.7%; *p* = 0.001). The AUC of IL-6 level at 1 h after ROSC, for predicting good neurological outcomes, was 0.59 (95% CI, 0.35–0.83; *p* = 0.47). The AUC of T_nadir_ was significantly superior to that of plasma IL-6 (*p* = 0.027, Figure 7).

### 3.4. Tnadir Was Associated with Alterations in Proinflammatory Cytokines in the Brain Immediately after ROSC

**Figure 8:**
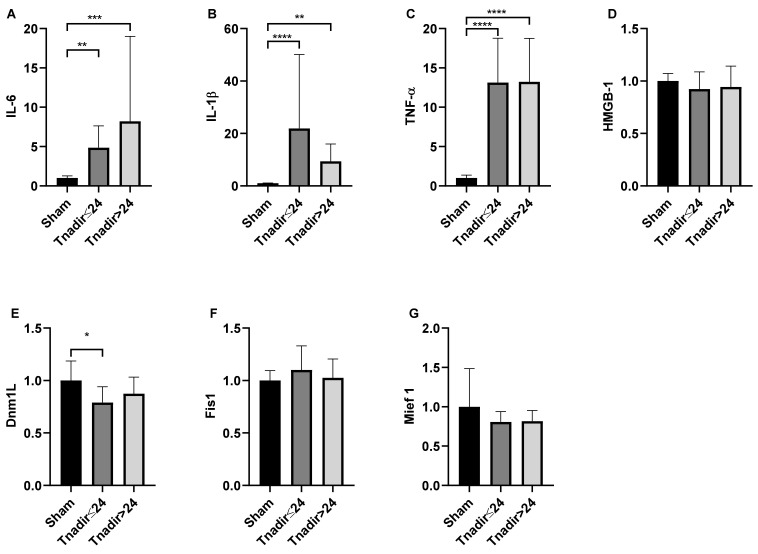
The associations between T_nadir_ and biomarkers in the post-cardiac arrest brain. The levels of (**A**) interleukin (IL)-6, (**B**) IL-1β, (**C**) tumor necrosis factor (TNF) α, (**D**) high-mobility group box 1 (HMGB-1), (**E**) dynamin 1-like protein (Dnm1L), (**F**) fission 1 protein (Fis 1), and (**G**) mitochondrial elongation factor 1 (Mief 1) in the brains of sham-operated rats and rats 2 h post return of spontaneous circulation. Data are presented as mean ± SD. *n* = 7, 10, and 21 in sham, T_nadir_ ≤ 24 min, and T_nadir_ > 24 min groups, respectively; * *p* < 0.05, ** *p* < 0.01, *** *p* < 0.001, **** *p* < 0.0001.

It has been reported that excessive cytokine production and dysfunctional mitochondrial dynamics are negatively associated with neurological outcomes after CA [28,31,32,33]. In the brain 2 h post-ROSC, cardiac arrest and CPR led to an increase in the mRNA levels of IL-6, IL-1β, and TNF-α, but not HMGB-1 (Figure 8). However, in comparisons between animals with T_nadir_ ≤ 24 min and with T_nadir_ > 24 min, there was no significant difference in IL-6 (*p* = 0.99), IL-1β (*p* = 0.44), and TNF-α (*p* = 0.99). A significant difference in Dnm1L gene expression was observed between sham animals and those with T_nadir_ ≤ 24 min, but not between animals with T_nadir_ ≤ 24 min and T_nadir_ > 24 min (*p* = 0.38). These observations suggest that T_nadir_ is not associated with cytokine productions and mitochondrial fission genes in the brain at 2 h after ROSC.

### 3.5. T_nadir_ Was Associated with Neuronal Degeneration Immediately after ROSC

**Figure 9:**
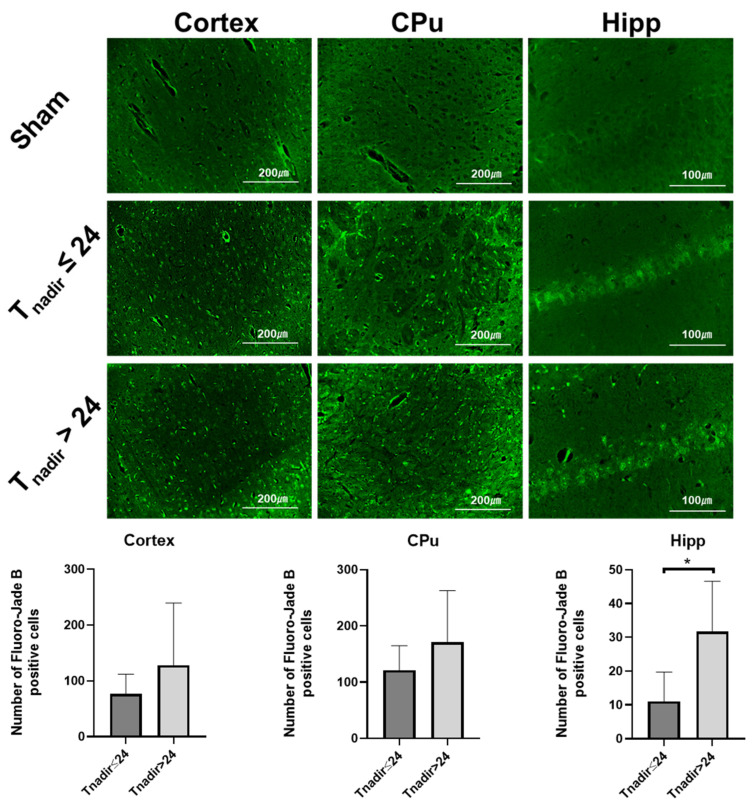
Fluoro-Jade B (FJB) immunostaining in the cortex, caudoputamen, and hippocampus. (Upper) Representative photomicrographs of brain sections (cortex, caudoputamen (CPu), and hippocampus (Hipp) CA1 region), showing FJB-positive cells in sham-operated rats and post-cardiac arrest rats having T_nadir_ ≤ 24 min and Tnadir > 24 min. (Lower) Number of FJB-positive cells in the cortex, caudoputamen, and hippocampus regions in survived rats having T_nadir_ ≤ 24 min (*n* = 9) and Tnadir > 24 min (*n* = 6). Data are presented as mean ± SD. * *p* < 0.05.

To further elucidate the predictive role of T_nadir_ on global brain damage after CA, we evaluated neuronal degeneration in the cerebral cortex, hippocampus, and caudoputamen by FJB immunostaining. FJB-positive degenerating neurons were conspicuous in animals with T_nadir_ > 24 min, whereas animals with T_nadir_ ≤ 24 min showed significantly fewer degenerating neurons than those with T_nadir_ > 24 min in the hippocampus region (Figure 9). These observations suggest that T_nadir_, measured within an hour after ROSC, was associated with degenerating neurons in the hippocampus region of the brain 72 h after ROSC.

## 4. Discussion

In the present study, we demonstrated that a longer CA duration resulted in delayed electrical recovery in the brain and greater degree of alterations in cerebral oxygen metabolism post-ROSC. To the best of our knowledge, this is the first study to demonstrate that the temporal changes in rSO_2_ shortly after ROSC were associated with severity of brain damage after CA/CPR in rats. There were no significant differences in rSO_2_ values, over 2 h after ROSC, between good and poor neurological outcome groups. However, T_nadir_ predicted good neurological outcome at 72 h after ROSC. T_nadir_ is a novel definition we found and means the time from ROSC to the nadir value. Based on the AUC analysis, T_nadir_ appears to be an excellent prognostic marker with significant advantages over plasma IL-6. Furthermore, T_nadir_ ≤ 24 min was significantly associated with lower neuronal degeneration in the hippocampus.

NIRS has long offered promise in monitoring the course of resuscitation after cardiac arrest and has remained inconclusive. A key impediment of rSO_2_ has been lack of a reliable scale of magnitude, which is susceptible to variation in device placement and subject anatomy. Since most clinical NIRS devices assume a venous/arterial distribution in the cerebral cortical tissue as 70/30% or 75/25% [34], rSO_2_ values are primarily influenced by cerebral venous oxygen saturation [35]. Monitoring of rSO_2_, during resuscitation in CA patients, has attracted attention because it can be a useful measure to predict the probability of ROSC and neurological outcomes [7,8,9,10,11,12,13]. However, previous systematic reviews and meta-analyses did not reveal any definite rSO_2_ cutoff values to predict ROSC and good neurological outcome [7,8,36,37,38]. Given that the absolute values and/or different degrees of variability in rSO_2_, due to several factors, vary among NIRS devices under various conditions [37,38,39,40], it will be challenging to clarify the definite cutoff value that can predict outcomes in patients with CA. In this regard, previous studies have suggested that relative changes in rSO_2_ from baseline are more appropriate for guiding resuscitative efforts than absolute values [7,9,10,11,12,13].

The present study demonstrates that T_nadir_ had a significant negative correlation with favorable neurological outcomes, with a cutoff value of 24 min, predicting a good neurological outcome, with an area under the receiver operating characteristic curve of 0.881. The exact mechanism of rapid decrease in rSO_2_, shortly after ROSC in rats with good neurological outcome, remains unknown. However, given the earlier recovery of EEG activity and a tendency of higher oxygen consumption immediately after ROSC in the lower severity model (6 min CA), it can be speculated that impaired neural activity and metabolism shortly after ROSC may affect the time-dependent changes in rSO_2_. Furthermore, our results showed that T_nadir_ was not associated with 72 h survival. Taken together, T_nadir_ can only predict brain injury but not mortality, which is clinically relevant for the prognostication of neurological recovery in the management of patients resuscitated from CA.

In the present study, the NIRS sensor has been designed to measure the rSO_2_ value at a depth of up to 10 mm from the attached skin, which corresponds to the depth of the hippocampus in rats. The group with T_nadir_ ≤ 24 min, which had a good neurological outcome, showed less neuronal degeneration (as assessed by FJB staining in the hippocampal CA1 region but not in the cerebral cortex or caudoputamen) than rats with T_nadir_ > 24 min. These observations suggest that in our rat model, rSO_2_ values reflect the corresponding area of interest in the brain.

The excess release of circulating cytokines, such as ILs and TNF, mediates ischemia/reperfusion injury, brain dysfunction, and myocardial dysfunction, following CA [6]. Consistent with these observations, in our study, the plasma IL-6 levels increased significantly 1 h after ROSC, compared to the baseline. Previous studies have shown that high levels of inflammatory cytokines are associated with mortality and/or poor neurological outcomes [28,31,32,41]. We demonstrated that AUC was significantly higher for T_nadir_ than plasma IL-6 1 h post-ROSC, suggesting that non-invasive, continuous measurements of rSO_2_ are more useful in the very early phase after ROSC than the known biomarker. However, we failed to show that plasma IL-6 level was a reliable predictor of neurological outcome and mortality. The discrepancy from the previous report [28,31,32,41] may have resulted from the early sample collection time in the present study. Moreover, the present study showed that inflammatory markers (IL-6, TNF-α, IL-1β) and mitochondrial fission markers in the brain 2 h post-ROSC did not differ between animals with T_nadir_ ≤ 24 min and T_nadir_ > 24 min. Further studies are warranted to determine whether the systemic inflammatory response and mitochondrial dynamics are associated with the severity of post-CA brain injury.

This study had some limitations. First, the relationship between rSO_2_ and oxygen consumption needs confirmatory studies, using more invasive measurements. However, since our idea with T_nadir_ evaluates the difference in temporal location of an inflection point rather than an absolute value, it is not necessary to confirm the consistency between the two parameters. Second, the site of sensor attachment can potentially influence the measured rSO_2_ value because of the difference in arterial and venous distributions of each rat. We ensured sensor attachment to the same site in all experiments, to preserve accuracy of measurements. Third, we did not examine the exact causes of death after CA; therefore, the underlying reason for the inability of T_nadir_ to predict survival outcome remains unknown. Fourth, cerebral hemodynamic parameters (transcranial Doppler or cardiac output) were not assessed in conjunction with changes in rSO_2_ values; however, a combination of assessments with these parameters could have improved the understanding of cerebral hemodynamic disturbances. Fifth, a lack of rigorous CPR technique (constant rate, measured depth of chest compression) in a non-blinded manner could introduce potential bias for brain recovery after CA. Finally, the animals were mechanically ventilated with 100% O_2_ for 105 min after ROSC, which may have contributed to increased inflammatory markers and mortality rate. To minimize experimental variations, both groups were subjected to the same oxygen administrations throughout the experiment.

## 5. Conclusions

We have discovered a novel metric, using inflections during rSO_2_ monitoring, called T_nadir_. The present study showed that T_nadir_ is a novel marker for predicting good neurological outcomes. The calculation of T_nadir_ in an individual may be useful for predicting neurological outcomes and tailoring the treatment in patients who resuscitated from CA, even if it is difficult to calculate the cutoff value that applies to all CA cases.

## Figures and Tables

**Table 1:** Baseline characteristics and perioperative parameters between good and poor neurological outcome groups.

	Poor Neurological Outcome Group (*n* = 21)	Good Neurological Outcome Group (*n* = 9)	*p* Value
Weight, g	441.0 ± 31.5	432.7 ± 27.6	0.50
MAP baseline, mmHg	87.7 ± 13.2	82.0 ± 17.0	0.12
HR baseline, bpm	294.4 ± 35.4	262.6 ± 24.5	0.02
BT baseline, °C	36.4 ± 0.2	36.6 ± 0.2	0.004
EtCO_2_ baseline, mmHg	38.9 ± 4.0	39.8 ± 2.9	0.29
rSO_2_ value baseline, %	66.6 ± 2.1	66.4 ± 1.9	0.77
Time to CA, s	186.3 ± 22.7	165.9 ± 26.9	0.04
Time to ROSC, s	80.1 ± 22.5	67.7 ± 9.0	0.13
MAP 20 min after ROSC, mmHg	123.2 ± 23.7	116.0 ± 14.2	0.41
HR 20 min after ROSC, bpm	335.1 ± 44.2	390.1 ± 39.4	0.003
BT 20 min after ROSC,	36.8 ± 0.3	36.8 ± 0.3	0.69
EtCO_2_ 20 min after ROSC, mmHg	40.5 ± 4.1	43.0 ± 5.9	0.19
rSO_2_ value 20 min after ROSC, %	62.6 ± 5.3	61.0 ± 2.1	0.05
MAP 90 min after ROSC, mmHg	101.2 ± 12.9	108.4 ± 8.6	0.14
HR 90 min after ROSC, bpm	331.4 ± 35.7	354.4 ± 50.8	0.17
BT 90 min after ROSC,	36.7 ± 0.3	36.7 ± 0.4	0.96
EtCO_2_ 90 min after ROSC, mmHg	38.7 ± 10.1	37.6 ± 4.4	0.66
rSO_2_ value 90 min after ROSC, %	60.8 ± 2.8	62.7 ± 2.5	0.09

MAP, mean arterial pressure; HR, heart rate; BT, body temperature; EtCO_2_, end tidal carbon dioxide; rSO_2_, regional cerebral oxygen saturation; CA, cardiac arrest.

## Data Availability

The datasets used and/or analyzed during the current study are available from the corresponding author on reasonable request.

## Supplementary Materials

The following are available online at https://www.mdpi.com/article/10.3390/jcm11010131/s1. Table S1: the assay IDs and primer sequences for each gene. Table S2: baseline characteristics of animals among the three different CA duration groups.
