# Supplementary material for: Real-Time Brain Monitoring by Near-Infrared Spectroscopy Predicts Neurological Outcome after Cardiac Arrest and Resuscitation in Rats: A Proof of Concept Study of a Novel Prognostic Measure after Cardiac Arrest
Source: J Clin Med. 2021 Dec 27;11(1):131. doi: 10.3390/jcm11010131 (PMC8745661; doi:10.3390/jcm11010131)
Supplement: Supplementary file 1 [file jcm-11-00131-s001.zip › jcm-1503073-supplementary.pdf]

Journal of
Clinical Medicine

MDPI

Table S1. The assay IDs and primer sequences for each gene.

Gene Name	Assay ID	Primer Sequence
IL-1b	Rn00580432_m1	
IL-6	Rn01410330_m1	
HMGB1	Rn02377062_g1	
DNM1L	Rn00586466_m1	
FIS1	Rn01480911_m1	
MIEF1	Rn01502709_m1	
Gapdh	Rn01749022_g1	
TNF-α	Custom Assay	Forward Primer: CCAGGAGAAAGTCAGCCTCCT<br>Reverse Primer: TCATACCAGGGCTTGAGCTCA<br>Probe Sequence: AGAGCCCTT-GCCCTAAGGACACCCCT

IL, interleukin; HMGB, high mobility group box protein; DNM1L, dynamin 1 like protein; FIS1, mitochondrial fission 1 protein; MIEF1, mitochondrial elongation factor 1 protein; TNF, tumor necrosis factor.

Table S2. Baseline characteristics of animals among the three different CA duration groups.

	6 min CA (n = 10)	9 min CA (n = 10)	12 min CA (n = 10)	*p* Value
Weight, g	444.9 ± 31.3	432.4 ± 26.9	438.2 ± 33.7	0.67
MAP baseline, mmHg	87.5 ± 16.0	86.4 ± 11.9	84.0 ± 16.1	0.86
HR baseline, bpm	265.5 ± 26.7	289.5 ± 22.3	299.6 ± 46.2	0.08
BT baseline, °C	36.5 ± 0.2	36.5 ± 0.3	36.3 ± 0.2	0.07
$EtCO_2$ baseline, mmHg	39.0 ± 2.8	38.3 ± 3.4	40.1 ± 4.7	0.56
rSO_2 value baseline, %	66.6 ± 1.9	66.1 ± 1.3	67.0 ± 2.7	0.62
Time to CA, sec	180.2 ± 33.4	188.9 ± 19.4	171.5 ± 20.5	0.32
Time to ROSC, sec	66.6 ± 9.0	75.1 ± 10.1	87.4 ± 29.7	0.08

MAP, mean arterial pressure; HR, heart rate; BT, body temperature; $EtCO_2$, end tidal carbon dioxide; rSO_2, regional cerebral oxygen saturation; CA, cardiac arrest. Three groups were compared by the one-way analysis of variance.
